# Beyond the nation-state: Anchoring supranational institutions in international business research

**DOI:** 10.1057/s41267-022-00537-3

**Published:** 2022-06-27

**Authors:** Simon Hartmann, Thomas Lindner, Jakob Müllner, Jonas Puck

**Affiliations:** 1 Institute for International Business, Vienna University of Economics and Business, Wien, Austria; 2 International Management Group, Institute for Strategic Management, Marketing, and Tourism, University of Innsbruck, Innsbruck, Austria

**Keywords:** institutional theory, multinational corporations, international organizations, supranational institutions

## Abstract

In this paper, we broaden the conceptualization of institutions beyond the nation-state and develop the concept of an institutional triality that represents two national institutional environments and the supranational level. While much of international business (IB) research has focused on national institutions and the differences between them, little attention and theorizing has been dedicated to the supranational institutions that form an integral part of the global institutional ecosystem. First, we conduct an interdisciplinary bibliometric survey on supranational institutions and a qualitative review of the central publications. Second, we initiate theory building and conceptualize supranational institutions from an IB point of view, distinguish them from related IB concepts, and discuss the conceptual properties of supranational institutions that are most relevant for MNCs. Building on these distinctions and properties, we provide a conceptual framework for understanding supranational institutions in the institutional triality. Third, we provide avenues for IB research building on the delineations, the conceptual properties, and the conceptual framework.

**Supplementary Information:**

The online version contains supplementary material available at 10.1057/s41267-022-00537-3.

## Résumé

Dans cet article, nous élargissons la conceptualisation des institutions au-delà de l'État-nation et développons le concept de trialité institutionnelle qui représente deux environnements institutionnels nationaux et le niveau supranational. Alors qu'une grande partie de la recherche en affaires internationales (International Business - IB) s'est focalisée sur les institutions nationales et les différences entre elles, peu d'attention et d’effort de théorisation sont consacrés aux institutions supranationales qui font partie intégrante de l'écosystème institutionnel global. Dans un premier temps, nous réalisons une enquête bibliométrique interdisciplinaire sur les institutions supranationales et une revue qualitative des principales publications. Ensuite, nous commençons la construction théorique et conceptualisons les institutions supranationales dans une perspective de l'IB. Nous les distinguons des concepts connexes de l'IB et discutons des propriétés conceptuelles des institutions supranationales qui sont les plus pertinentes pour les entreprises multinationales. Nous appuyant sur ces distinctions et ces propriétés, nous apportons un cadre conceptuel permettant de comprendre les institutions supranationales dans la trialité institutionnelle. Enfin, nous proposons des pistes de recherche en IB en partant sur les délimitations, les propriétés conceptuelles et le cadre conceptuel.

## Resumen

En este trabajo ampliamos la conceptualización de las instituciones más allá del estado nación y desarrollamos el concepto de trialidad institucional que representa dos entornos institucionales nacionales y el nivel supranacional. Mientras que mucha de la investigación en negocios internacionales se ha enfocado en las instituciones nacionales y las diferencias entre ellas, poca atención y teorización se ha dedicado a las instituciones supranacionales que forman parte integral del ecosistema institucional global. Primero, llevamos a cabo un sondeo bibliométrico interdisciplinario sobre las instituciones supranacionales y una revisión cualitativa de las publicaciones centrales. Segundo, iniciamos una construcción teórica y a conceptualizar las instituciones supranacionales desde el punto de vista de negocios internacionales, diferenciándolas de otros conceptos relacionados en negocios internacionales, y discutimos las propiedades conceptuales de las instituciones supranacionales que son más relevantes para las empresas multinacionales. En base en estas diferencias y propiedades, proporcionamos un marco conceptual para entender las instituciones supranacionales en la trialidad instituciona. Tercero, proporcionamos posibilidades para la investigación en negocios internacionales sobre las delimitaciones, las propiedades conceptuales, y el marco conceptual.

## Resumo

Neste artigo, ampliamos a conceituação de instituições para além do estado-nação e desenvolvemos o conceito de uma trialidade institucional que representa dois ambientes institucionais nacionais e o nível supranacional. Embora grande parte da pesquisa em negócios internacionais (IB) tenha se concentrado em instituições nacionais e nas diferenças entre elas, pouca atenção e teorização são dedicadas às instituições supranacionais que formam uma parte intrínseca do ecossistema institucional global. Primeiramente, realizamos um levantamento bibliométrico interdisciplinar em instituições supranacionais e uma revisão qualitativa das publicações centrais. Em segundo lugar, iniciamos a construção de teoria e conceituamos instituições supranacionais sob o ponto de vista de IB, distinguimo-las de conceitos relacionados a IB e discutimos as propriedades conceituais de instituições supranacionais que são mais relevantes para MNCs. Com base nessas diferenças e propriedades, fornecemos um modelo conceitual para entender as instituições supranacionais na trialidade institucional. Terceiro, fornecemos caminhos para construção de pesquisa em IB sobre delineações, propriedades conceituais e modelo conceitual.

## Abstract (zh)

在本文中, 我们拓宽了民族国家之外的制度概念, 并提出了代表两个国家制度环境和超国家层面的制度三隅对称性概念。尽管大部分国际商务 (IB) 研究都集中在国家制度及它们之间的差异上, 构成全球制度生态系统组成部分的超国家制度很少被关注和理论化。首先, 我们对超国家制度进行跨学科文献计量调查, 并对核心出版物进行定性综述。其次, 我们从 IB 角度对超国家制度启动理论建设和概念化, 将它们与相关的 IB 概念区分开来, 并讨论与跨国公司最相关的超国家制度的概念属性。基于这些区分和属性, 我们提供了一个概念框架来理解制度三隅对称性中的超国家制度。第三, 我们为基于描述、概念属性和概念框架的 IB 研究提供了途径。

## INTRODUCTION

Understanding how multinational corporations (MNCs) navigate cross-border institutional complexity has been a constituting mandate of international business (IB) scholarship (Kostova & Roth, 2002; Sun, Doh, Rajwani, & Siegel, 2021). MNCs investing in foreign countries with weak institutions face “institutional voids” within these national institutional environments (Khanna, Palepu, & Sinha, 2005; Peng, Wang, & Yi, 2008) that, for example, can result in higher uncertainty, liabilities of foreignness, or increased costs of operations. Once MNCs operate in multiple national institutional environments, they are exposed to “institutional duality” where home- and host-country national institutions may exert potentially opposing pressures on MNCs (Kostova & Roth, 2002; Nell, Puck, & Heidenreich, 2015). Despite its rich contributions to institutional literature, IB research has mostly conceptualized institutions on a single (e.g., voids or institutional quality) or dyadic country level (e.g., institutional distance).^1^ Pinkham and Peng (2017) refer to this theoretical assumption as ‘one law, one court, in one country’.^2^ The focus on (pairs of) countries, however, ignores the existence and relevance of supranational institutions, which co-exist with national institutions and can add an additional, superordinate layer to the national institutional contexts, and their differences. IB research has made some inroads into understanding supranational institutions (e.g., Jandhyala & Weiner, 2014; Nebus & Rufin, 2010; Ramamurti, 2001), but a structured distinction from national institutions, and a conceptual understanding of what characterizes supranational institutions, is still missing. In this paper, we investigate supranational institutions as a theoretically important, yet often overlooked, dimension of MNCs’ institutional ecosystems.

The lack of recognition of how supranational institutions are conceptually distinct entities in IB research is relevant for three reasons. First, such institutions have been subject to lively research in other disciplines such as political science (e.g., Booth, 2011; Büthe & Milner, 2008; Cafruny, 2001; Fukuyama, 2014; Kerner & Lawrence, 2014; Voeten, 2019) and economics (e.g., Bagwell & Staiger, 2006; Rodrik, 2018a; Rogoff, 1999; Stiglitz, 2002). Further, many of the effects of supranational institutions identified in these disciplines bear strong implications for core IB topics and MNC activities such as FDI, exporting, political risk management, and location choice. For example, Baker, Bloom, and Davis (2016) link the creation and demise of supranational institutions to economic policy uncertainty, which in turn is central to many IB questions. Hence, it is surprising that IB research has not yet drawn on insights into supranational institutions from other disciplines or attempted to develop a systematic definition or understanding of supranational institutions from an IB perspective.^3^ At the same time, hardly any effort has been made by other disciplines to fertilize the academic discourse with the rich institutional perspectives in IB.

Second, supranational institutions are not mere extensions of national institutions. Instead, we argue that supranational institutions have some idiosyncratic properties that are practically relevant to MNCs. Advancing IB understanding, a conceptualization of supranational institutions, a clear distinction from other IB-relevant concepts, and a systematic discussion of the properties and effects of supranational institutions requires theorizing about the complex interactions within the institutional triality. In our conceptualization, this triality represents the complex institutional environment considering home-country institutions, host-country institutions, and supranational institutions that MNCs interact with when they engage in cross-border business. So far, IB research lacks a clear understanding of which circumstances supranational institutions function under as substitutes or supplements for national institutions (e.g., Büthe & Milner, 2008; Lubell, Henry, & McCoy, 2010; Neumayer & Spess, 2005; Tobin & Rose-Ackerman, 2011), when they reinforce or conflict with national institutions (e.g., Hoffman, 1999; Moore, Brandl, & Dau, 2021), and how these interactions depend on the national context.

Third, the dearth of research on supranational institutions in IB is worrying because of their immense importance in tackling the Grand Challenges of International Business (e.g., Buckley, Doh, & Benischke, 2017). For example, past decades have seen emerging supranational institutions in the areas of climate change, human rights, and poverty reduction (e.g., UN Sustainable Development Goals). Properly incorporating such supranational institutions requires IB scholars to adjust their institutional perspectives to a ‘post-national world order’ (Scherer & Palazzo, 2011). At the same time, however, some developed countries have seen tendencies of de-globalization (Witt, 2019) and a return of nationalist and protectionist politics. The resulting national-level political movements (e.g., Trumpism, Brexiteers) often conflict with supranational institutions. This leads to ambiguity and uncertainty for firms doing business in these environments and creates a need to understand the complex interactions between the national and supranational spheres in which MNCs operate.

In this paper, we provide an impetus towards an understanding of the supranational institutional framework in three steps. First, we take stock of the status quo of interdisciplinary research on supranational institutions using quantitative and qualitative review methods. We provide a bird’s-eye view of the immense research on supranational institutions across all academic disciplines by conducting a bibliometric analysis of 44,812 scholarly publications in the Web of Science. Our goal is to gain the broadest possible overview of research that self-identifies as supra-institutional in some form.^4^ To do this, we simplistically extend the understanding of national institutions in IB to the supranational level. From this perspective, we identify different types of supranational institutions and topics associated with them. We then narrow our focus and analyze a selection of MNC-related contributions within the disciplines of international business and management, economics, and political science.

Second, we initiate theory building on supranational institutions based on observations from our literature review. We thereby develop an understanding of what supranational institutions are and how they differ from other core IB concepts such as national institutions, institutional distance, and shared history. We identify six properties of supranational institutions that emerge as important for understanding how MNCs can navigate the supranational institutional environment: supraterritoriality, co-existence, selectivity, contextuality, consensuality, and co-evolution. Each property points to specific theoretical implications and recommendations for IB research. Then, we consolidate the distinctions between national and supranational institutions in conceptualizing how MNCs and supranational institutions interact.

Third, we outline the most promising research gaps for IB literature and suggest avenues for future research that may help provide a deeper understanding of how MNCs might navigate their institutional environments. Doing so, we suggest improving the conceptual understanding of the types and characteristics of formal and informal supranational institutions and how supranational institutions differentially affect MNCs. In addition, we identify the need for research to understand the co-evolution of MNCs and supranational institutions, including the genesis of supranational institutions. IB research will also benefit from explicitly investigating the interplay between supranational institutions and other between-country concepts, such as institutional distance. Finally, we suggest a better interdisciplinary exchange between IB, political science, and economics research.

This stepwise approach to theory building contributes to IB research in four ways. First, we review the body of academic literature from multiple disciplines, providing an interdisciplinary overview of the literature on supranational institutions. Second, we provide a set of characteristics of supranational institutions helping IB research conceptualize them and distinguish supranational institutions from other important IB concepts. Third, we initiate theory building on the relationships between national institutions, supranational institutions, and firms in an institutional triality, and suggest how this interaction may shape international strategy through IB theory. Fourth, we suggest avenues for future research that will better link supranational institutions to firms’ international strategies and investigate how research in IB and beyond can understand the creation and proliferation of supranational institutions.

## SUPRANATIONAL INSTITUTIONS AS EXTENSIONS OF NATIONAL INSTITUTIONS

The first major challenge of reviewing research on supranational institutions is that such institutions affect so many aspects of social and economic activity that their study extends across many disciplines, often with very different definitions. Within individual disciplines, supranational research is often scattered. To capture supranational institutions in a broad sense, and to initiate interdisciplinary theorizing, a broad view applying an intentionally broad definition is a helpful start. We take a broad definition to begin with in this quantitative overview, and we zoom in on how supranational institutions are relevant for IB once we have a broad overview.

There are many ways to define institutions, and much controversy (e.g., Glaeser, La Porta, Lopez-de-Silanes, & Shleifer, 2004; Hodgson, 2006; Searle, 2005). Two scholars – William R. Scott and Douglass C. North – are particularly influential and widely acclaimed (and cited) representatives of institutional theory in IB, economics, and political science. To obtain the broadest possible but still widely accepted definition, we therefore begin with their definitions of institutions. According to Scott (1995: 56), “institutions compromise regulative, normative, and cultural-cognitive elements that, together with associated activities and resources, provide stability and meaning to social life.”^5^ Relatedly, North (1994: 360) understands institutions as “humanly devised constraints that structure human interaction. They are made up of formal constraints (e.g., rules, laws, constitutions), informal constraints (e.g., norms of behavior, conventions, self-imposed codes of conduct), and their enforcement characteristics. Together, they define the incentive structure of societies and specifically economies.”^6^

These definitions have important commonalities. First, both assume that institutions are structural, and that in order to be meaningful they need actors to interpret them and, in doing so, provide them meaning. An important consequence is the necessity to keep institutions, the ‘rules of the game’ (e.g., rules, norms, beliefs), distinct from the ‘players’ of the game (e.g., individuals, organizations). A good example is laws as an institution, where individuals abiding by laws, police, judges, and fellow citizens are all potential ‘players’ and provide meaning to these institutions. Second, both Scott and North state that institutions are ‘multifaceted’ and encompass formal rules, informal norms, and cognitive elements such as beliefs (North, 1994: 363; North, 2005; Scott, 1995: 68). The behavior of players is shaped not only by laws, regulations, and policies but also by codes of conduct, traditions, principles, and standards rooted in history and culture. An important consequence here is that institutions are overlapping, and at times reinforcing or conflicting. One example is that norms and beliefs are recurrently identified as a major determinant of formal institutions and that, if they are misaligned with norms and beliefs of societies, they become ‘dead law’ (Berkowitz, Pistor, & Richard, 2003; Rodrik, 2008; Seidler, 2018).

Based on these commonalities, in our attempt to grasp supranational institutions, we focus on institutions as ‘rules, norms, and beliefs’ (both formal and informal) and discuss them in relation to ‘players’ (e.g., governments, international organizations, MNCs). Although the definitions by Scott and North are commonly used in studies of institutions on the country level, we argue that they extend beyond the nation-state. In a globally connected world, national institutions are not independent of each other. Rather, they co-exist in a global institutional ecosystem in which supranational social structures govern and moderate the relationships between national institutions and MNCs (Sun et al., 2021). Thus, ‘rules’ become ‘supranational rules’, like for example GATT articles or Investor-State Dispute Settlement (ISDS) procedures. The ‘norms and beliefs of behavior’ extend to ‘supranational norms and beliefs of behavior’, which include historically developed codes of conduct, standards, and traditions. The role of ‘players’ includes actions like sanctions and military interventions that are usually conducted by powerful actors (e.g., powerful governments like the US, or MNCs like Apple Inc.). Just like national institutions, the supranational institutional ecosystem comprises multifaceted institutions (both formal and informal), but the defining element of supranational institutions is that their governing effect transcends national institutional borders.

Based on these commonalities and on the works of North and Scott, we broadly define supranational institutions as “supranational rules, norms, and beliefs, which structure interactions among individuals and organizations” for the purpose of taking stock of the interdisciplinary academic literature.

## BIBLIOMETRIC REVIEW PROCESS

Building on the broad definition of supranational institutions we provide above, we begin our review of the literature on supranational institutions with a bibliometric assessment. In this quantitative overview, we follow prior literature in the field (e.g., Lindner, Puck, & Doh, 2021; Nerur, Rasheed, & Natarajan, 2008) with the intent to identify topics central to research on supranational institutions and to link those to important conceptualizations of institutions. We use co-citation analysis (Zupic & Čater, 2015) and co-word analysis to quantitatively analyze the body of literature (Aria & Cuccurullo, 2017).

To achieve this bird’s-eye view, we build a search string based on the understanding of supranational institutions developed above. First, we define search terms that reflect the ‘beyond the nation-state’ scope of institutions based on formal supranational institutions (rules and synonyms^7^ of rules) and informal supranational institutions. Each synonym for formal and informal institutions is combined with five different synonyms for supranational: ‘international’, ‘transnational’, ‘global’, ‘bilateral’, and ‘multilateral’. Details about the process of synonym creation are available from the authors upon request.

The search strategy yields a total of 44,812 contributions in the Web of Science Database (accessed June 7, 2021). It is important to stress that, by nature of the search string, the sample includes only literature that self-identifies as supranational AND institutional in some way. This, we believe, is appropriate for the bibliometric review that provides an overview of explicitly supranational institutional literature.^8^

Research on the topic of supranational institutions has increased dramatically from 1956 to today, with an output of approximately 100 articles per year up to the 1970s, approximately 200 per year until the 1990s, more than 1000 each year beginning in 2009, and more than 3000 articles published in 2020. Only a tiny fraction (0.3%) of the literature on supranational institutions was published in IB journals (as defined by Tüselmann, Sinkovics, & Pishchulov, 2016), providing justification for our broad definition and interdisciplinary approach. Search results yield papers from fields as diverse as medicine, fishery, astronomy, and computer science. Supranational institutions play important roles in these research fields because they govern cross-country issues within related industries and professions. However, these specialized fields typically lack the theoretical underpinning in institutional theory necessary to understand supranational institutions in a way similar to IB literature.

Despite the breadth of research disciplines, however, the most important topics discussed in the body of 44,812 papers cover many areas central to IB literature. Among the most prominent keywords are ‘global governance’ (663 articles), ‘globalization’ (537), ‘China’ (579), and ‘governance’ (405). Papers published outside traditional IB journals but using these IB-relevant keywords substantially outnumber those using the same keywords that were published in traditional IB journals. This indicates that research published in non-IB publications may bear findings relevant to IB. In addition, the most prominent topics in the body of literature, but again published mostly outside of IB journals, are ‘climate change’ (1342 articles) and ‘global warming’ (1001), which IB scholars have identified as Grand Challenges for the field (e.g., Buckley et al., 2017).

**Figure 1:**
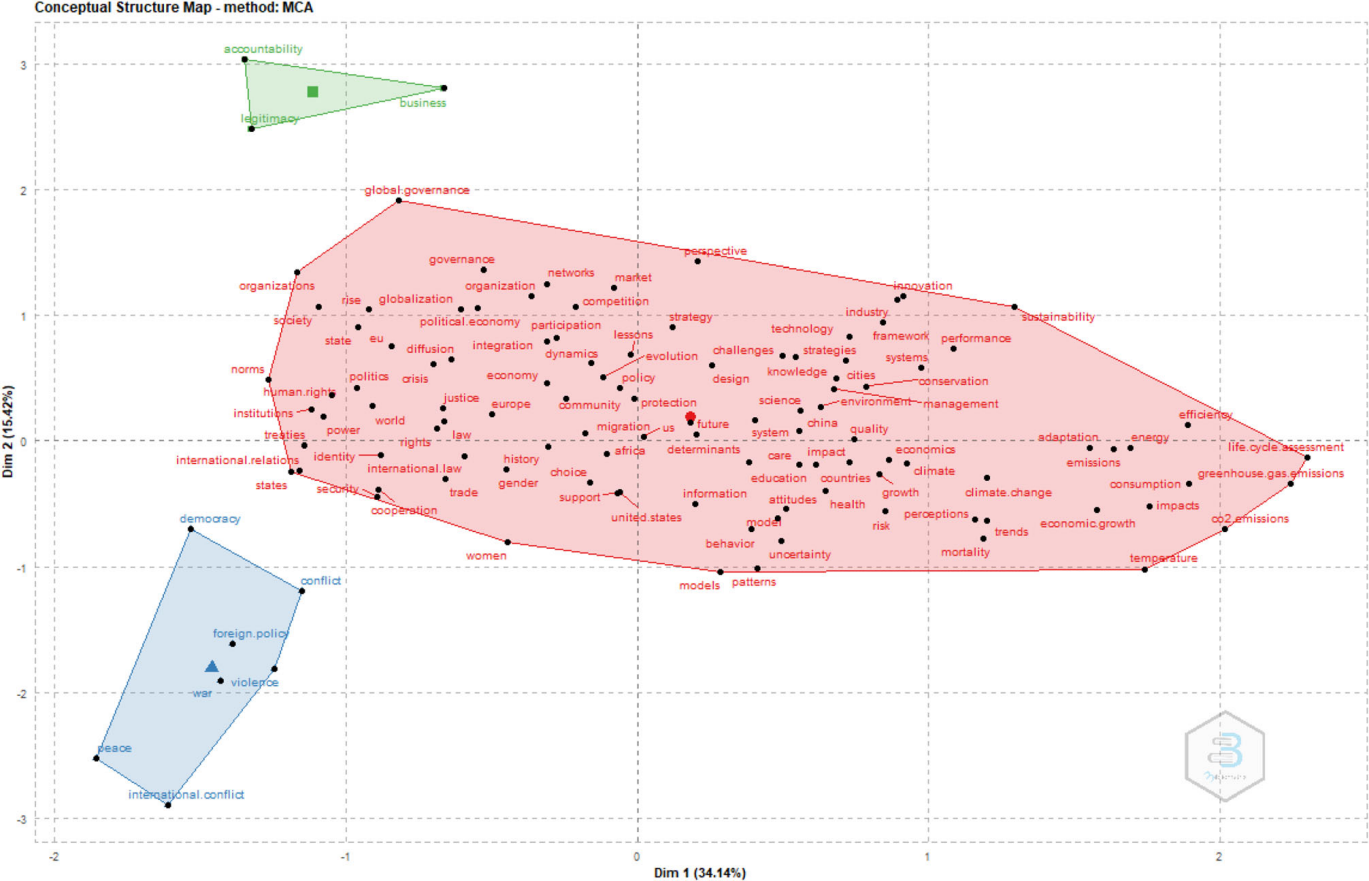
Conceptual structure of literature on supranational institutions.

Moving the analysis one step forward, we conduct a co-word analysis to categorize the keywords in individual papers by topical cluster (see Appendix 1 for methodological details about how this analysis is conducted). Results of the multiple correspondence analysis (Figure 1) show three distinguishable clusters in the body of literature on supranational institutions.^9^ The largest cluster (red, focusing largely on human development and public welfare) combines a broad range of topics including ‘human rights’, ‘health’, ‘economic growth’, and ‘climate’. The blue cluster covers topics related to relationships between countries like ‘foreign policy’, ‘war’, ‘peace’, and ‘international conflict’. These topic areas have been less of a focus in IB because of their predominant societal, as opposed to business, implications. Nevertheless, topics in the red and blue clusters are related to policy decisions affecting IB and could provide some interesting MNC-level research.^10^ The green cluster broadly addresses topics around ‘business’, ‘legitimacy’, and ‘accountability’. These topics are most closely related to the activities of multinational firms.^11^

**Figure 2:**
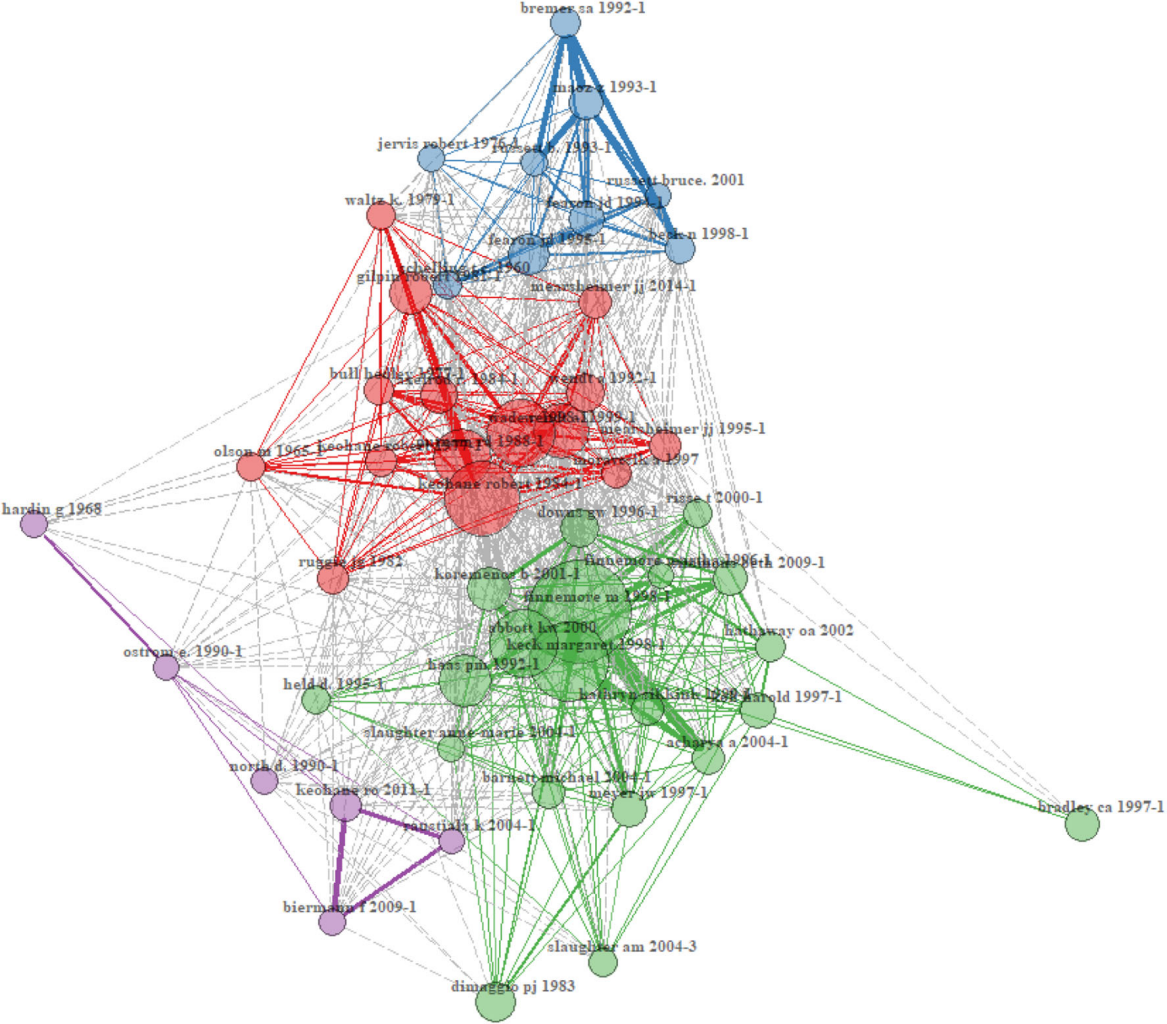
Core references in the literature on supranational institutions.

To understand the theoretical foundations of the literature on supranational institutions, we retrieve co-citation frequencies for the 50 most cited articles (see Appendix 2 for methodological details regarding the co-citation analysis). Figure 2 reveals four clusters in the literature that form the conceptual foundation for the body of literature.^12^ Solid lines in Figure 2 represent co-citation links to articles in the same cluster; dashed lines indicate co-citation links to articles in other clusters. Bubble size indicates the overall citation counts of the respective articles, and line strength indicates the number of co-citations.^13^

Our analysis of the 50 most important references in Figure 2 reveals a structure similar to the topic areas identified in Figure 1 and enables us to link research fields to the most influential researchers. The green cluster of foundational literature in Figure 2 is particularly central to recent IB literature on institutions. Specifically, this cluster contains influential work on institutions (most prominently, DiMaggio & Powell, 1983). The pink cluster, which has strong links to both the green and the red clusters, also contains important foundational literature on the role of institutions in global business and includes some of the foundations of new institutional economics (North, 1990). Applying the categorization proposed by Kostova, Beugelsdijk, Scott, Kunst, Chua, and van Essen (2020), the green and the pink clusters connect with two streams of institutional theory in IB: organizational institutionalism (green) and institutional economics (pink).

Bringing together the insights from our bibliometric analysis of 44,812 papers on supranational institutions highlights two salient gaps that require further exploration in IB literature and draws an important conclusion about how we can use the breadth of literature to develop a meaningful definition of supranational institutions for IB theory. First, our analysis of research topics (Figure 1) reveals blind spots in the discussion of supranational literature within IB journals. IB research published in traditional IB journals is focused on an important, but limited, subset of the overall literature on supranational institutions. The clustering into a separate group of topics regarding the business environment in the green cluster points to topics central to IB (as represented by keywords along the lines of ‘legitimacy’ and ‘business’) and reveals a separate stream of literature distinct from the other two streams addressing challenges such as social development and climate change. While there is a substantial overlap of key topics and keywords (particularly centering on global governance and the growing importance of China), there is much more to supranational institutions (e.g., regarding their role in facilitating sustainable development and foreign policy) than the IB literature currently covers.

Second, our analysis of research underlying the literature on supranational institutions (Figure 2) reveals that the different academic disciplines in the social sciences (e.g., economics, political science, IB) have quite separate intellectual cores and, as a result, theoretical conceptualizations of these institutions. However, institutional theory seems to be relevant across disciplines, and the Kostova et al. (2020) distinction of schools of thought of institutional theory appears to exist across fields, although often implicitly. Nevertheless, the exact understanding of what (supranational) institutions are, and how they interact with MNCs, is quite diverse.

Given the breadth of literature identified in this quantitative literature review, we believe that IB research requires a more specific definition of supranational institutions than may result from the simplistic extension of Scott’s and North’s understanding of national institutions to the supranational level. The very diversity of literature that emerged from this naïve extension of the most common definitions of national institutions in IB shows that more conceptualization and theory building are necessary. In order to initiate theorizing from an IB perspective, we move from the quantitative overview of topics and strands of literature to a more detailed and qualitative review of the various types of supranational institutions that were discussed in research self-identifying as relevant in the context of supranational institutions.

## REVIEW RESULTS

The objective of this qualitative review is to tighten the focus of the quantitative literature overview and to develop a conceptual understanding of what supranational institutions are within IB, political science, and economics. Particularly, we are interested in understanding, in a structured and systematic way, how supranational institutions relate to MNCs.^14^ To achieve this deeper focus, we reduce the extensive body of literature of 44,812 papers in all disciplines to a manageable number in three steps. In short, we reduce articles to those allocated to the management and business, economics, and political science fields, and then we limit the body of literature to articles investigating how MNCs relate to supranational institutions, and, finally, we add papers prominently cited in the body of literature. For details regarding this selection mechanism and a list of the most recent related publications in JIBS, please see online Appendix 3.

Supranational institutions discussed in IB literature are diverse and include both formal and informal institutions – though authors rarely explicitly distinguish between formal and informal supranational institutions. On the most formal side of supranational institutions, IB research has historically focused on formal agreements between countries. IB scholars have shown, for example, that formal supranational institutions like investment protection agreements (Jandhyala & Weiner, 2014), bilateral trade agreements (Jandhyala, Henisz, & Mansfield, 2011), and intellectual property protection agreements (Brandl, Darendeli, & Mudambi, 2019), along with international arbitration proceedings (Devarakonda, Klijn, Reuer, & Duplat, 2021; Pinkham & Peng, 2017), can fill institutional voids in host countries and mitigate political risk, because supranational institutions constrain national institutional players (e.g., the host-country government). Political science and economics share an interest in international agreements like bilateral investment treaties, preferential trade agreements, and double-taxation agreements (Barthel & Neumayer, 2012; Büthe & Milner, 2008; Tobin & Rose-Ackerman, 2011), but emphasize more the limits to legalization and enforcement of these treaties and agreements through third parties (e.g., Abebe & Ginsburg, 2019) instead of the benefits of these treaties for MNCs in facilitating trade and investment across national borders.

Some supranational institutions discussed in IB are somewhat codified but lack consistent ratification as laws or regulations. In most such cases, pressure to comply comes from collective actions, boycotts, and sanctions from a diverse set of players. As a result, MNCs voluntarily comply or associate with such predominantly informal institutions. In some cases, national regulators can refer to these standards, best practices, and guidelines and give them local legitimacy and third-party enforcement.^15^ IB scholars have studied such predominantly informal supranational institutions in corporate social responsibility (Christmann, 2004; Christmann & Taylor, 2006; Gardberg & Fombrun, 2006; Husted & Allen, 2006; King, Lenox, & Terlaak, 2005), financial reporting standards (Barkemeyer, Preuss, & Lee, 2015; Fortanier, Kolk, & Pinkse, 2011), ISO certification (Bansal & Bogner, 2002; Goedhuys & Sleuwaegen, 2016; Rondinelli & Vastag, 1996), standard-setting (Haeussler & Rake, 2017), climate-change initiatives (Kolk, Levy, & Pinkse, 2008; Pinkse & Kolk, 2012), poverty reduction (Kolk, Rivera-Santos, & Rufín, 2018), labor standards in global supply chains (Donaghey, Reinecke, Niforou, & Lawson, 2014), corruption (Darrough, 2010), and sustainable development practices (Kolk, 2016). Though these predominantly informal institutions lack ratification as laws or regulations, they are commonly promoted by supranational institutional players (e.g., intergovernmental organizations). Thus, they do not have third-party enforcement but influence MNCs’ cross-border activity through organized collective action. Ingram, Robinson, and Busch (2005) and Alcacer and Ingram (2013), for example, show empirically that joint membership in supranational players (intergovernmental organizations; IGOs) helps bridge institutional distance between countries and encourages both trade and FDI between countries and, as a consequence, firm internationalization.

IB scholars have also studied a wide array of supranational informal institutions that are not codified, nor actively promoted, nor enforced by supranational players. These institutions gain their institutional effect purely from uncoordinated activities through shared values that are not restricted to a national territory. In this category of institutions, the distinction between national institutions, institutional distance, and supranational institutions becomes blurred, and references to the supranational nature of institutions are often implicit only. Authors have, for example, studied the institutional constraints that result from countries’ (and MNCs’) embeddedness in the broader economic and geopolitical context of nations. Nebus and Rufin (2010), Gould and Winters (2007), Henisz (2011), Müllner and Puck (2018), and Ramamurti (2001), for example, provide perspectives on MNC–state bargaining in which the bargaining power of a national government is constrained by its socio-economic and political embeddedness (Kobrin, 2001a, b, 2017; Vernon, 1968, 1971). Relatedly, some research has also studied MNC strategies in financial markets to reduce political risk in host countries (Dorobantu & Müllner, 2019; Dorobantu, Lindner, & Müllner, 2020; Fotak, Lee, & Megginson, 2019; Hainz & Kleimeier, 2012; Maggi, 1999) and how membership in trade agreements influences how firms manage risk (e.g., Oh & Oetzel, 2017). Other supranational institutions studied in this area are historically evolved immigrant networks and diaspora communities in IB (De Lange, 2013; Hernandez, 2014; Hernandez & Kulchina, 2020; Jandhyala & Phene, 2015; Kunczer, Lindner, & Puck, 2019; Li, Hernandez, & Gwon, 2019).

A smaller number of studies in our review apply the idea of supranational institutions to investigate values, norms, and beliefs that are shared not by a national population but by a supranational demographic. For example, some researchers have promoted the idea of a global civil society and even a type of transnational culture (Asmussen & Fosfuri, 2019; Castells, 2008; Gould & Grein, 2009). Such supranational institutions, it is argued, earn their institutional status not from enforcement but through issue-based networks of like-minded interconnected individuals and organizations.^16^ Players in such institutions are transnational networks of activists (Boddewyn & Doh, 2011), social movements (Morgan, 2007), and NGOs (De Lange, Armanios, Delgado-Ceballos, & Sandhu, 2016; Teegen, Doh, & Vachani, 2004; Waddock, Bodwell, & Graves, 2002).

Economists share this view on the competitive nature of global networks (Babic, Garcia-Bernardo, & Heemskerk, 2020; Seabrooke, & Wigan, 2017), whereas political science scholars focus more on cooperative elements between players on the supranational level (e.g., Chilton, Milner, & Tingley, 2020; Ross & Voeten, 2016; Urpelainen, 2010) and the interaction of cooperation and competition (e.g., Wellhausen, 2019). Moreover, both disciplines share an interest in the inner workings of supranational institutions and how their effective governance is driven by the roles of diverse private and public initiatives (Kahler, 2016; Libecap, 2014; Ostrom, Burger, Field, Norgaard, & Policansky, 1999). Specifically, migrant networks hold great interest for economists because the former have the potential to promote economic integration across nations while also potentially diverting foreign investments because of prejudice (Fossati, 2019). Another shared interest between IB and economics is the view of supranational institutions as private efforts to supplement or counter inefficient or ill-functioning national institutions (e.g., Johns, Thrall, & Wellhausen, 2020; Posner, 2010; Talukdar & Meisner, 2001). These contributions are particularly important because they stress the active role of private individuals and companies in the genesis and proliferation of supranational institutions rather than focusing entirely on the passive effect of supranational institutions to govern private cross-national activity.

Aside from these shared interests with existing IB literature on supranational institutions, distinct types of informal supranational institutions appear in the political science and economics literature. These include economic sanctions (Lektzian & Biglaiser, 2013; Mirkina, 2018; Mityakov, Tang, & Tsui, 2013; Muchlinski, 2001) and supranational initiatives tackling environmental concerns (Bayer, Marcoux, & Urpelainen, 2015; Collins & Thomas, 2016; Onishi, 2007). Development assistance via technology-sharing policies (Di Vita, 2013; Hoekman, Maskus, & Saggi, 2005), as another example, is almost exclusively discussed by economists.

Table 1 summarizes some of the most important commonalities and differences between how supranational institutions are understood across the disciplines included in our bibliometric analysis and qualitative review. The table highlights that disciplines share the acknowledgement that supranational institutions are different from national institutions. There is also agreement that supranational institutions concern the cross-national activities of MNCs and nations, whereby the different disciplines focus on different elements, as reflected in the clusters of our bibliometric review of the literature.

**Table 1:** Commonalities and disciplinary specificities across disciplines

	IB	Political science	Economics
Commonalities in topics and studied institutions	SI are different from national institutions, but the dimensions of the differences vary across disciplines. Acknowledgement of the effect of SIs in regulating cross-border relationships and transactions. The active role of the private sector in the creation and proliferation of SIs. The role of SIs in addressing the Grand Challenges.		
Disciplinary foci and emphasis (simplified)	Selective topics primarily within the business cluster (e.g., legitimacy). The effect of SIs in cross-border private transactions (firm level). Strategic use of formal SIs by MNCs to fill institutional voids. Antecedents of MNCs adherence to predominantly informal SIs	Broader universe of topics across all clusters (e.g., human rights). The role of SIs in International Relations on governments and society. The determinants of supranational institutions. Cooperative SIs and the role of authority and expertise (e.g., cooperation to regulate externalities on supranational level).	Broader universe of topics across all clusters (e.g., development). The role of SIs in economic development. Functioning, enforcement and limitations of SIs. Competitive SIs and the role of incentives (e.g., state versus private power in global value chains).
Most prominently studied supranational institutions and players	Formal (e.g., BITS), informal (e.g., voluntary standards), players: MNCs	Formal (e.g., international agreements, human rights), informal (e.g., political and civil society initiatives), players: governments, administration, civil society, transnational movements	Formal (e.g., GATT , FTA, BITs, DTAs, environmental agreements), informal (global networks, private solutions), players: WTO, firms, NGOs, transnational movements

The table offers a stylized simplified representation. Abbrev.: Supranational institution (SI)

Particularly in economics and political science, there is a strong recognition of the active role of the private sector in creating and proliferating supranational institutions. This role is particularly important in addressing the Grand Challenges (Buckley et al., 2017) that are difficult to govern nationally. While IB historically studies the influence and strategic value of supranational institutions for firms, political scientists and economists focus more on the boundary conditions that supranational institutions create in the interaction between national institutions. Similarly, IB focuses on how players organize in response to supranational rules, norms, and beliefs; the other disciplines focus more on the impact of MNCs on supranational institutions. As a result, political science and economics have also generated more research on the limitations and inner functioning of supranational institutions than IB research.

## THEORETICAL EXTENSION AND INTEGRATION

The interdisciplinary bibliometric analysis and the qualitative review of MNC-related contributions on supranational institutions unveil many angles taken in research on supranational institutions. We find remarkable heterogeneity in the views and priorities in this body of research. Whereas IB research, for example, provides rich insights into the top-down effects of supranational institutions on MNCs, the other disciplines focus more on the bottom-up view. Also, IB research typically begins from the notion that formal and informal institutions exist, and then proceeds to study their implications for MNCs. The other disciplines are more concerned with making sense of the evolution (political science) of supranational institutions and how they can effectively be enforced (economics). In addition, none of the three disciplines have an overview or review paper providing an overarching definition of supranational institutions.

However, what defines a supranational institution appears to be mostly implicit, and we see no clear pattern within and across disciplines. Because of the breadth of approaches in the literature, we take a step-wise approach to building a conceptual overview helping IB research further understand supranational institutions. First, we delineate supranational institutions from related, but theoretically distinct, constructs in IB research. Second, we build on this delineation and suggest which properties of supranational institutions are critical for IB research. Third, we provide a conceptual framework for understanding supranational institutions as a component of the institutional triality, including how supranational institutions interact with MNCs.

### What Supranational Institutions are Not

To initiate theory building in IB, we first distinguish supranational institutions from other important IB concepts. Three concepts require specific attention. First, we build on the somewhat naïve definition of supranational institutions that we provide in the lead-up to the quantitative review, and we provide a more complete distinction of supranational from national institutions. Second, we discuss what differentiates supranational institutions from institutional distance because variation in distance may be driven by joint influence from supranational institutions. Third, we distinguish supranational institutions from shared historical ties because literature has argued that shared historical ties and associated dependence relationships may also lead to shared characteristics between national institutional environments, which are hard to differentiate from supranational institutions.

First, all three streams of literature in the review highlight that supranational institutions co-exist with national institutions but are not restricted to a national territory. Supranational institutions can influence MNCs operating in several countries in a superordinate way (Jandhyala & Weiner, 2014; Kostova & Zaheer, 1999; Sun et al., 2021), either through superordinate rules or through norms and beliefs shared across countries. When supranational institutions matter in a transaction, a two-tiered institutional triality, consisting of two national institutional contexts plus the supranational level, is created.^17^ For example, a multilateral investment treaty exists in addition to, and largely independent of, the national institutional contexts. This characteristic is pivotal in that it implies that a supranational institution, from an IB perspective, is only truly a supranational institution if it is a tertiary institutional force that exists in addition to the national institutional environments. In this co-existence, economics and political science literature has established that supranational institutions either supplement or conflict with national institutions.

Second, supranational institutions are distinct from institutional distance. In IB research, economic subjects come from different national institutional environments, which creates difficulties in cross-border transactions (Beugelsdijk, Ambos, & Nell, 2018; Kostova et al., 2020).^18^ These differences can be quantified – as is often done in empirical IB literature – through differences in national characteristics (e.g., economic development, political systems). Similarities between national institutional settings, however, do not constitute a supranational institution if they do not result from the influence of a superordinate set of rules, norms, or beliefs. For transactions involving substantial influence from supranational institutions, the distance/difference between national and supranational institutions will also matter, particularly if there is an asymmetry in the influence of supranational institutions on the home and host countries. As a result, when supranational institutions are involved, MNCs and IB researchers might have to consider three sets of distances, between the two home-country national institutions and between each national institution and the supranational level.

Third, IB can benefit from differentiating between supranational institutions and shared history or economic dependence. Research has documented the stabilizing effects of shared history through military alliances (Li, Arikan, Shenkar, & Arikan, 2020; Li & Vashchilko, 2010), colonial ties (Bertrand, Betschinger, & Settles, 2016), immigrant networks (Almeida, Phene, & Li, 2015; Balachandran & Hernandez, 2021), and diaspora communities (De Lange, 2013; Hernandez, 2014; Inouye, Joshi, Hemmatian, & Robinson, 2020). Other research has proposed similar arguments for economic embeddedness and power (Barnett & Duvall, 2005; Hurd, 1999; Rangan & Sengul, 2009; Wade, 2011). Clearly, economic history and the economic relationships that two countries have accumulated in the past will have shaped both national institutions and their distances. Further, historical ties may also, directly or indirectly, lead to the development of supranational institutions relevant to both nations (e.g., bilateral trade agreements between two countries). However, we argue that shared history and economic dependence by themselves are not supranational institutions, because they are not superordinate and do not exist independently (i.e., even in the absence of one of the countries involved) of the involved national institutional settings.

To summarize, the main distinctions between supranational institutions and other important IB concepts like national institutions, institutional distance, and shared history are that supranational institutions add a layer on national institutions or an institutional triality, which often alters how national institutions affect MNCs. Note that our distinctions are made from an IB perspective in an effort to initiate theory building. Thus, these may not be equally relevant in other disciplines.

### Conceptual Properties of Supranational Institutions from an IB Perspective

After distinguishing supranational institutions from other important IB concepts, we distil the conceptual properties of supranational institutions that are most important from an IB perspective. We identify six such properties, building on the findings from the reviews conducted above: supraterritoriality, co-existence, contextuality, selectivity, consensuality, and co-evolution.

First, supranational institutions have *supraterritorial* influence on MNCs: they can affect an MNC that is native to another national institutional environment without having a direct political legitimization in the respective country. The key contingency of supraterritoriality is enforcement. In the case of most formal institutions, supraterritoriality is unambiguously determined in a Westphalian sense (Habermas, 2001; Scherer & Palazzo, 2011), through the coercive legal enforcement of agreements and contracts in a particular country. In the case of informal institutions, supraterritoriality depends on the outreach of the players that collectively enforce supranational norms (e.g., global activist campaigns).^19^ Supraterritoriality consequently implies that institutional IB research should reflect on relevant formal and informal supranational institutions that could directly affect their research subjects at home and abroad. Supraterritoriality also means that some supranational institutions can exert influence or be enforced outside of bi- or multilateral institutional settings. In an extreme example of such supraterritorial enforcement, a global outrage by media, NGOs, and labor rights experts in response to the Rana Plaza collapse in Dhaka in 2013 forced garment producers, as well as firms in their supply chains, to comply with safety standards that far outstrip national rules and regulations.

Second, supranational institutions *co-exist* with national institutions in an institutional triality. This implies that IB research needs to account for both national and supranational institutional components. Focusing only on national institutions and their differences, or only on supranational institutions, will inevitably omit important elements of the institutional envelope (Ahuja, Capron, Lenox, & Yao, 2018). Co-existence of supranational institutions with national institutional environments highlights the question of how MNCs can navigate the partially conflicting institutional triality. As discussed above, supranational institutions, on occasion, bridge institutional differences^20^ and may reduce institutional distances (Sun et al., 2021). Co-existence also means that supranational institutions can supplement or conflict with national institutions (Neumayer & Spess, 2005; Tobin & Rose-Ackerman, 2011). When supplementing national institutions, supranational institutions can help fill institutional voids. When they conflict with national institutions, this may create challenging situations similar to the institutional duality described by Kostova and Roth (2002) but in a triadic form.^21^

Third, political science research has highlighted that the effectiveness of supranational institutions is *contextual* and depends on lower-order national institutional environments. For example, different national institutions mean that MNCs from different countries may have different exposures to the same supranational institutional pressures. Contextuality may result, for example, from national governments actively shielding companies from formal supranational institutions by impeding the enforcement of formal supranational institutions^22^ or by promoting opposing values and norms against supranational norms.^23^ When national institutions shield firms, supranational institutions may influence MNCs in at least two ways. First, supranational institutions may influence MNCs in the country directly, by providing rules, norms, or beliefs in areas where national institutions do not. Second, supranational institutions can pressure MNCs’ foreign assets, partners, and customers through their influence in other institutional environments. In addition to intentional shielding through national governments, contextuality may also result unintentionally from a lack of complementary players on the national level for enforcement of supranational institutions (Saiger, 2020; Yackee, 2008). For example, most formal supranational institutions lack sovereign status and thus executive powers. Consequently, effective enforcement requires functioning national institutions and players (courts, police, etc.). For IB research, contextuality means that the study of top-down effects of supranational institutions on MNCs must account for specific country-level contingencies. Findings on the effects of specific supranational institutions on MNCs do not necessarily generalize to other countries.^24^

Fourth, supranational institutions *selectively* influence firms within a national institutional setting. Depending on their international operations, firms investing in different countries are not equally and equitably affected by the same supranational institution. As a result, such institutions may lead to selective advantages for some firms over others. For example, many international investment agreements are negotiated and signed between specific nations and thus potentially discriminate geographically. Even within a specific national dyad, eligibility clauses can determine whether specific investments by MNCs are eligible for protection under the agreement.^25^ Some BITs have been argued to disadvantage domestic firms relative to foreign MNCs given that foreign firms enjoy supranational investor protection (e.g., through investor-state dispute settlement, ISDS). Domestic companies are not protected in the same way from their national governments’ bargaining under ISDS (Dixit, 2011; Tobin & Rose-Ackerman, 2011). Another particularly controversial practice that results directly from the selectivity of formal supranational institutions is jurisdiction shopping, in which MNCs use the selectivity of supranational institutions to build an institutional advantage over competitors (Ahuja & Yayavaram, 2011). When it comes to informal supranational institutions, some MNCs may be more targeted for violating supranational norms by activists than others because of their visibility (Eesley, Decelles, & Lenox, 2016). As a result, they may voluntarily choose to adhere to, or associate with, supranational institutions (e.g., Equator Principles, ISO Standards) while others do not. For IB research, selectivity poses the challenge of additional firm-level complexity but also provides an opportunity to study the strategic value of supranational institutions and the challenges they pose for MNCs.

Fifth, supranational institutions require *consent* of agents with sufficient power for enforcement because supranational institutions lack national sovereignty. Consensual enforcement can occur on the highest level of aggregation with two national institutional players (e.g., governments) agreeing on a nationally ratified and enforced supranational institution (e.g., a bilateral investment treaty) that subsequently affects MNCs in their transactions (Devarakonda et al., 2021; Moore et al., 2021; Pinkham & Peng, 2017). In supranational informal institutions, consensus can occur between a sufficiently large number of players or even between individuals (often across many countries) capable of exerting pressure on MNCs (e.g., civil society, international activism). Consensus between the two transacting parties is sometimes sufficient to make a supranational institution applicable to a transaction. When, for example, negotiating business transactions between two firms that do not share a language, the parties can agree on using a common language or lingua franca. English, which has evolved into a supranational lingua franca, presents an example for such a mutually agreed supranational institution.

Sixth, supranational institutions, national institutions, and MNCs *co-evolve* endogenously. Through broad acceptance and consensus, national institutions can inspire or turn into a supranational one. Co-evolution also means that MNCs are simultaneously tributaries, advocates, and architects of supranational institutions. On the one hand, supranational institutions constrain MNCs in their international operations. On the other hand, MNCs can contribute to the development of supranational institutions (Boddewyn & Brewer, 1994). Co-evolution also applies to national institutions (Cantwell, Dunning, & Lundan, 2010; Jackson & Deeg, 2019), but through a different mechanism. In a supranational context, the process of institution-building relies much more on multinational collaboration with peer firms and other players (e.g., civil society) than in national institutional evolution. MNCs can shape supranational institutions in many ways. For example, they can organize collectively, often under the purview of supranational institutions or players (e.g., OECD, ILO), to build supranational institutions in the form of global public policy networks (Bartley, 2018; Danielsen, 2005; Detomasi, 2007). The Global Reporting Initiative (Barkemeyer et al., 2015), Carbon Disclosure Project (Kolk et al., 2008), Equator Principles (Contreras, Bos, & Kleimeier, 2019; O’Sullivan & O’Dwyer, 2015), and OECD Guidelines for Multinational Enterprises are examples of supranational institutions in which MNCs collaborate with supranational players to proliferate supranational rules, norms, and beliefs.^26^ Economic research in particular highlights MNCs’ efforts in transnational lobbying (e.g., Maggi, 2020) to shape the formal supranational ecosystem. IB research is uniquely positioned to study the broader role of MNCs in shaping supranational institutions and their influence on society. Thus, IB research should extend its theoretical horizon to include some of the supranational phenomena studied in related disciplines, such as commitment problems and enforcement (Biermann, Pattberg, Van Asselt, & Zelli, 2009; Chayes & Chayes, 1993; Fearon, 1998; Powell, 2006). Table 2 summarizes the six relevant conceptual properties of supranational institutions from an IB perspective.

**Table 2:** Relevant conceptual properties of supranational institutions

Property	Description	Consequence	Implications for MNCs
Supraterritoriality	Unlike national institutions, supranational institutions span national borders	Supranational institutions may be enforced in multiple, even extraterritorial countries (jurisdictions)	MNCs need to strategically account for supranational rules and norms between and beyond nation-states
Coexistence	Supranational institutions exist alongside national institutions but on a higher level	Supranational institutions directly affect national institutions or moderate distances between national institutional environments	MNCs have to be aware that supranational institutions alter, complement or substitute the effects of national institutions
Contextuality	Supranational institutions’ two-tiered effect on MNCs often depends on national institutions (e.g., imperfect enforcement, national shielding, insufficient capabilities)	The effect of supranational institutions may vary from one home country to the other	MNCs require analysis of how deeply supranational institutions target countries, as well as competition on economic and political markets on a macro level
Selectivity	Supranational institutions sometimes affect MNCs selectively (e.g., eligibility, voluntary adoption)	The direct effect of supranational institutions may vary within countries from one MNC to another	MNCs require analysis of how intensely supranational institutions affect themselves, their competitors, suppliers, and customers on a micro level
Consensuality	Lacking supranational enforcement, supranational institutions require consent either on the level of contracting parties or on a higher level that can enforce institutions vis-à-vis MNCs	Supranational institutions can be agreed on ad hoc by transacting parties or be enforced on them	MNCs need to consider how supranational institutions are enforced in a specific transaction context. MNCs can influence the consensus regarding enforcement on different levels (transaction, national, supranational) but with different means
Co-evolution	Supranational institutions co-evolve with national institutions and MNCs	MNCs are tributaries, advocates, and architects (“entrepreneurs”) of supranational institutions	MNCs can actively engage in developing supranational institutions (in alignment with strategic considerations), shaping supranational institutions to match MNC strategy

### Conceptual Framework

**Figure 3:**
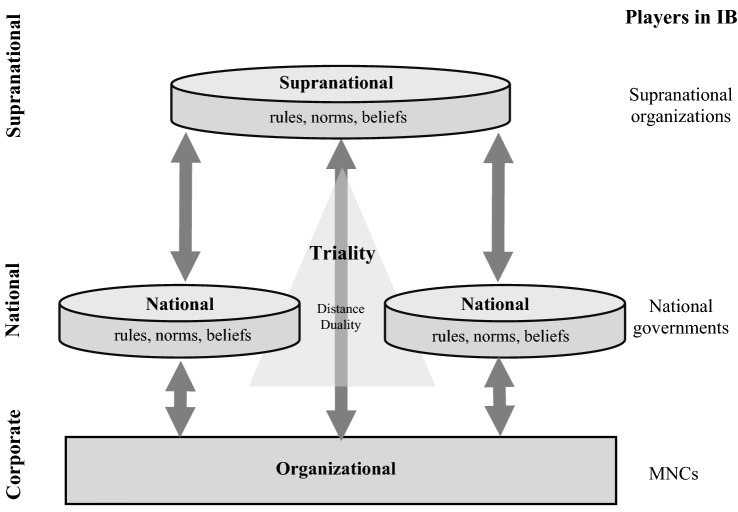
The institutional triality.

Bringing together the characteristics and relevant properties of supranational institutions, we next suggest a mechanism for how MNCs are influenced by and influence supranational institutions. The global ecosystem of institutions we propose has three levels: supranational, national, and organizational. Supranational institutions govern interactions between any combinations of the three.^27^ The influence of supranational institutions on MNCs may unfold directly (one-tiered) or through (combinations of) national institutions (two-tiered). In addition, the relationships are not unidirectional: MNCs may also influence supranational institutions from the bottom up. Thus, the global institutional ecosystem co-evolves in a dynamic, reciprocal process (Cantwell et al., 2010). Figure 3 provides a conceptual framework of the multi-tier institutional ecosystem (which we label “institutional triality”) and the relationships between institutions and players across different levels. We build this framework on the two-tiered MNC–state bargaining model proposed by Li, Newenham-Kahindi, Shapiro, and Chen (2013) to structure the discussion along the number of tiers (one-tier/two-tier) and directions (bottom-up/top-down).

Reaching across borders, MNCs are uniquely positioned to create and shape supranational institutions (Kostova & Zaheer, 1999), often referred to as ‘institutional entrepreneurship’. Hence, MNCs directly create supranational institutions (bottom-up, one-tiered). For example, financial market regulation through International Financial Reporting Standards (IFRS) represents a set of supranational rules and norms created by a company-driven body (the International Accounting Standards Board; IASB). Firms also create supranational institutions mediated through national institutions (bottom-up, two-tiered). The creation of technological standards, in, for example, the German Institute for Standardization (DIN), is typically driven by technical innovations at the firm level. Through certification by the DIN, these standards become national norms. To ease economic interaction across countries, the European Committee for Standardization (CEN) collects and integrates norms that emerged from national member organizations, creating supranational norms from firm initiatives in a two-tiered way.

Once created, supranational institutions may influence MNCs in a one- or two-tiered fashion. For them to matter for MNCs, such institutions require a form of enforcement. For formal supranational institutions (e.g., BITs or EU directives), enforcement typically relies on ratification into national law (top-down, two-tiered). As a result of national ratification, players on the national level also enforce supranational institutions. Informal supranational institutions do not necessarily require an explicit a priori consensus on a national level to constrain national and supranational players (top-down, one-tiered). It suffices that there is informal consensus between sufficiently powerful institutional players to impose supranational institutions on firms and individuals. For example, civil society or pressure groups can constrain local activities irrespective of domestic laws (e.g., outrage over Brazilian rainforest clearance, pollution, mining, work standards).

There is also some interdependency between the top-down and the bottom-up mechanisms connecting MNCs and supranational institutions. For example, the supranational IFRS also have substantial influence on national accounting rules and policies, in turn creating national institutions that MNCs have to comply with. Similarly, the top-down pressures resulting from EU directives on clean production, for example, will lead to technical innovations, which in turn may become supranational institutions through the standard-setting process described above. In order to provide a structure for IB research investigating the complex relationships between supranational and national institutions, as well as MNCs, we next suggest avenues for future research.

## OUTLOOK FOR IB RESEARCH

We suggest five broad research avenues for future theoretical and empirical IB research: (1) better understanding of the types of supranational institutions as well as (2) their differential effects on MNCs; (3) the genesis of supranational institutions including the co-evolution of MNCs and supranational institutions; (4) the interplay between supranational institutions and related concepts (such as national institutions, institutional distance, and shared history); and (5) the linking of institutional research in IB to related fields, particularly economics and political science.

First, IB research will benefit from a more nuanced understanding of the characteristics and types of supranational institutions (upper tier in Figure 3). Our quantitative review finds a multiplicity of diverse supranational institutions but no systematic classification. In addition, our bibliometric review points to topical blind spots within IB research with respect to how supranational institutions interact in IB on the Grand Challenges (e.g., human rights, migration, MNCs’ role in sustainable development, and foreign policy). Further, our quantitative and qualitative reviews also identified conceptual blind spots in the IB literature on supranational institutions. Other than in IB, research in economics and political science focusses strongly on the evolution of informal supranational institutions, especially the role of private initiatives in shaping and enforcing norms and values (Alcacer & Ingram, 2013; Ruggie, 2018). For example, the proliferation of ESG principles in the financial sector has altered the competitive landscape of the industry in an unprecedented way. These standards are imposed essentially by an international network of organizations, activists, and/or cultures instead of by national financial regulators (Contreras et al., 2019; O’Sullivan & O’Dwyer, 2015). In this example, it is the consensual adoption by players in several countries that drives the proliferation of supranational institutions. These informal supranational institutions have, so far, received little attention in IB research. IB theory on liabilities of foreignness (e.g., Luo & Mezias, 2002; Zaheer, 1995) may particularly benefit from considering the genesis of (informal) supranational institutions because shared rules and norms resulting from shared supranational institutions may limit costs MNCs incur because of a lack of legitimacy in foreign markets. In addition, IB could benefit from building on the knowledge of the inner functioning and limitations of supranational institutions in political science and economics.

Second, IB research could study more intensely the effects of supranational institutions on MNCs (downward arrows in Figure 3), including how the rules and norms imposed by supranational institutions are enforced by the relevant players. On an MNC level, IB research could study firm-level differences in the supporting and constraining effects of supranational institutions on MNCs. The emerging literature on the role of supranational institutions in firms’ nonmarket strategy offers an example of such efforts, but research on this topic remains largely conceptual (Doh, Lawton, & Rajwani, 2012; Doh, McGuire, & Ozaki, 2015; Dorobantu, Kaul, & Zelner, 2017; Sun et al., 2021). However, IB inquiry should not be limited to how supranational institutions relate to firm-level characteristics. Rather, it can consider the contextuality and selectivity of supranational institutions. In other words, the effects of such institutions on MNCs depend not only on MNC characteristics but also, following the two-tiered structure of the institutional ecosystem, on the characteristics of the national environment, because supranational institutions co-exist with national institutions. For example, IB could study which national institutional characteristics impede or enhance the efficiency of supranational institutions in their effects on MNCs. Along these lines, IB theory on location and internalization advantages may benefit from explicitly considering the relevance of supranational institutions for where firms locate production facilities to benefit from supranational institutions such as BITs, and how supranational institutions relate to when MNCs organizationally internalize foreign operations. Similarly, IB could study how national governments use supranational institutions to influence MNCs (Tihanyi, Aguilera, Heugens, Van Essen, Sauerwald, Duran, & Turturea, 2019), or the other way around. Enforcement of supranational institutions by different actors will likely also drive how the supraterritoriality of supranational institutions affects firms operating in different countries. IB and its rich history in the study of national institutions is uniquely positioned to research varying firm-level effects from different institutional configurations. Based on the nuanced understanding of supranational institutions, the effect of supranational institutions holds interesting strategic considerations for MNCs. Particularly, supranational informal institutions are potentially immediate constraints to MNCs (e.g., through initiatives such as boycotts). A better understanding of supranational informal institutions will also reveal how MNCs can successfully interact with civil society.

Third, IB research should dedicate more effort to studying the genesis, dynamics, and co-evolution of supranational institutions and the role of MNCs therein (upward arrows in Figure 3). The evolution of supranational institutions (e.g., Cantwell et al., 2010) and the role of institutional dynamics for MNCs (e.g., Putzhammer, Slangen, Puck, & Lindner, 2020) have received some attention in IB. Political science and economics, however, have been much more active in this regard and provide immense inspiration for future research on how supranational institutions evolve (e.g., Abbott, Green, & Keohane, 2016; Chayes & Chayes, 1993; Kaczmarek & Newman, 2011; Wagner, 2016). IB research on institutional entrepreneurship (e.g., Chakrabarty, 2009; Doh, Rodrigues, Saka-Helmhout, & Makhija, 2017) may benefit from extending the study of national institutions to supranational institutions. In addition, the global COVID-19 pandemic and the shock waves it has sent through the economic system have highlighted the need for further supranational coordination to increase the resilience of global supply chains and ensure sustainable development. Many of the supranational institutions providing the rules and regulations for this coordination (some of which are still to be born) will address the big questions and Grand Challenges (Buckley et al., 2017) to which IB research needs to contribute.

A particularly interesting research question for IB and MNCs is the de-evolution or dissolution of supranational institutions. While much of the literature and theory in IB has historically assumed the ever-increasing globalization and continuous involvement of supranational institutions, recent geopolitical events put into question some of these fundamental assumptions of IB theory (Buckley & Hashai, 2020). Recent years have evidenced an increase of de-globalization forces and an accompanying partial dissolution of formal supranational institutions (Ambos, Cesinger, Eggers, & Kraus, 2020; Witt, 2019). The Brexit vote and the resulting reduction in EU member states to 27 is one evident symptom of the de-globalization trend (Bloom, Bunn, Chen, Mizen, Smietanka, & Thwaites, 2019; Rosamond, 2016). Much less publicized, India, South Africa, and Bolivia have revoked and cancelled bilateral investment treaties in recent years and countries like Venezuela, Bolivia, and Ecuador have opted out of the International Centre for Settlement of Investment Disputes (IISD, 2012). The return to protectionism and the rising pressures towards a dismantling of supranational institutions have often been fueled by more informal national populist movements (Hartwell & Devinney, 2021; James & Vaaler, 2018; Rodrik, 2018b). In addition to such country-specific de-globalization forces, scholars in IB have diagnosed increasing globalization skepticism across developed countries (Buckley & Hashai, 2020; Butzbach, Fuller, & Schnyder, 2020; Cuervo-Cazurra, Doz, & Gaur, 2020). The protests surrounding the G7 meetings and the Occupy movement are variants of a shared phenomenon. Understanding how these phenomena affect supranational institutions and, as a consequence, international business activities by MNCs should be central to the IB research agenda.^28^

Fourth, we see much room for theory development in the overlaps of supranational institutions with related concepts, particularly institutional distance. Institutional distance plays a central role in IB research. Scholars in IB have explored institutional distance in research questions ranging from location choice (e.g., Dow, 2000; Kunczer et al., 2019), entry mode (Slangen & van Tulder, 2009) to choice of financing (e.g., Lindner, Muellner, & Puck, 2016), and human resource practices (Brock, Shenkar, Shoham, & Siscovick, 2008), among many others. The question how the different dimensions of institutional distance relates to the shared compliance with supranational institutions (or lack thereof) remains open, however. Economics research has used the shared adoption supranational institutions to identify relevant effects, but even the empirical insights generated have had little influence on research on institutional distance in IB.

Fifth, IB research can put more effort into the proliferation of its contributions into other disciplines. IB as a research field has investigated many aspects of the firm–government interactions from an institutional theory perspective. This provides an opportunity for other disciplines, which have predominantly focused on the country level without considering the roles of MNCs, to enrich their theoretical perspectives. Institutional theory is a pillar in all disciplines surveyed but the understanding of the different schools of thought is something we predominantly found in IB literature. As is evident from our quantitative literature review (particularly Figure 2), the variety of schools of thought is not substantially reflected in the intellectual cores of disciplines outside IB. We believe that political science and economic literature might benefit from a broader understanding of institutional theory, building on these different schools of thought.

## CONCLUSION

This paper makes a first step towards structurally understanding the roles of supranational institutions in IB. While IB research has a rich history of investigating national institutions, our quantitative and qualitative literature reviews suggest that the influence of supranationals on MNCs (and vice versa) is under-researched. We suggest that it is necessary for IB research to complement research into institutional contexts and distances with a superordinate layer of supranational institutions. To provide a basis for this stream of investigation, we proceed in three steps. First, we provide a quantitative overview of research in supranational institutions and condense the literature on supranational institutions in IB, economics, and political science in a qualitative literature review. Second, building on these analyses, we delineate supranational institutions from national institutions, institutional distance, and the shared history that a group of countries may have. Third, we suggest six conceptual properties that characterize supranational institutions. We close the theoretical integration of our findings with a conceptual framework highlighting the mechanics of how supranational institutions influence MNCs and how supranational institutions are enforced. Fourth, we suggest five research avenues that IB scholars may explore in order to further understand supranational institutions.

In the first step, we provide a somewhat-naïve definition of supranational institutions by extending a broad definition of national institutions to the supranational level. With a search string derived from this extension of national institutions, we identify 44,812 research articles from a broad set of fields (including fishery, medicine, and IB) that discuss supranational institutions in some way. In a bird’s-eye view on this literature, we analyze the research topics (Figure 1) and intellectual cores (Figure 2) of this body of research. What emerge are three clusters of research topics (Figure 1) covering topics around development, international relations, and business environment. In Figure 2, we show the distinct schools of thought in institutional theory that permeate the disciplines included in our quantitative review but are not sufficiently structurally distinguished. Building on this quantitative review, we narrow (see Appendix 3 for details about the methodology) the body of literature to research on how supranational institutions influence MNCs. At the same time, we focus on research in IB, economics, and political science (Table 1). This review of literature reveals that outside the formal/informal distinction, there is little common ground in how research defines supranational institutions. We provide an overview of the research topics and understanding of supranational institutions and players. However, we cannot identify a clear distinction of how literature consistently differentiates between supranational institutions and related concepts; nor does our literature review identify shared characteristics of supranational institutions that literature could use to build a common understanding of supranational institutions.

In the second step, we condense the findings from our literature review into theory building. We begin by delineating supranational institutions from similar concepts prominent in IB research: we (1) differentiate supranational institutions from national institutions and highlight the two-tiered institutional triality that MNCs must work with when operating abroad. In addition (2), we highlight how supranational institutions differ from institutional distance. Finally (3), we distinguish supranational institutions from shared history among countries. With these distinctions in place, we next suggest six conceptual properties that emerge from our qualitative review of literature on supranational institutions in IB, economics, and political science: supraterritoriality, co-existence, contextuality, selectivity, consensuality, and co-evolution (Table 2). Having clarified shared characteristics of supranational institutions, we provide a framework conceptualizing the interaction of supranational institutions, national institutions, and MNCs (Figure 3).

In the third step, we combine the theory-building efforts with the main results from the quantitative and qualitative literature reviews and suggest five avenues for research that IB scholars can explore to further the understanding of how supranational institutions influence MNCs. First, IB research will benefit from a better understanding of different types of supranational institutions. Second, IB research needs to better understand the effects of supranational institutions on MNCs. Third, IB research can contribute to better understanding the co-evolution of MNCs and supranational institutions as well as the recent trends towards weakening supranational institutions. Fourth, IB research will profit from a clearer distinction of supranational institutions from institutional distance and the resulting differential effects on MNCs. Fifth, IB research may also contribute to progress in other fields, particularly by sharing with political science and economics research the deep understanding of how different schools of thought conceptualize institutions.

In sum, this review paper contributes to IB research in four ways. First, we provide structured quantitative and qualitative overviews of the massive multidisciplinary research on supranational institutions. Second, we suggest six characteristics idiosyncratic of supranational institutions. In doing so, we provide a starting point for theorizing on the specific effects of supranational institutions on MNCs. Third, we relate supranational institutions to national institutions and provide a conceptual framework for how the two types of institutions relate to MNC strategy. Fourth, we provide the first insights into how MNCs can co-create supranational institutions and how MNCs and supranational institutions influence each other.

## Notes

1. This is most likely due to IB’s historical origins studying MNCs’ internationalization as stepwise cross-border transactions with clearly defined home and target companies.
2. In a reflection on the role of business in global politics, Scherer and Palazzo (2011) borrow from Habermas (2001) and describe the transition from a Westphalian world order of nation-states to a post-national governance constellation characterized by a loss of state authority, increasing ambiguity of national borders, and an increase in transnational private governance.
3. In the recent special issue of the *Journal of International Business Studies* on the Long-Term Energy Transition and IB, for example, several articles study the role of supranational norms, regulations and organizations, and yet none of the articles engages in a conceptual discussion of supranational institutions and their influence on MNCs.
4. This view of the supranational ecosystems relates to the configurational view of institutions proposed by (Jackson & Deeg, 2019) but focuses more strongly on the relationship aspect between different institutions and layers of institutions.
5. Formal rules are typically enforced by third parties (e.g., courts, private arbitrators) whereas informal norms and beliefs of behavior are enforced by peers (North, 2005). Following Scott (1995), we also distinguish between regulative (coercion via legal sanctions), normative (social obligations via shared moral understanding), and cognitive institutions (shared beliefs), depending on how institutions relate to compliance, legitimacy, and order.
6. Similar as indicated in the definition of Scott, Douglass North has more recently emphasized the importance of the cognitive dimensions of rules and the role of beliefs in constituting institutions (North, 2005) and the role of violence (North, Wallis, & Weingast, 2009).
7. We derived the synonyms from the definition of institutions in the previous chapter. It is common that examples for rules are laws, regulations, polity etc., while synonyms like traditions, standards, values, etc., are used equivalent for norms and beliefs of behavior. This is also consistent with our reading of seminal literature on supranational institutions, which often use these or similar synonyms. The final list was compiled from this reading of the literature and a final brainstorming in an interdisciplinary team of colleagues.
8. In the subsequent qualitative review and the theoretical discussion, we follow the process of Sun et al. (2021), and also allow the inclusion of other contributions that do not self-identify explicitly as ‘supranational’.
9. Because we reduce the full keyword list to only papers with at least 150 mentions in the 44,812 papers, the clusters in Figure 1 only show a selection of topics covered in the literature on supranational institutions.
10. One controversial issue in the intersection of military conflict and private business is the role of private security contractors in military conflicts.
11. The clusters are derived from shared keywords between papers. Naturally, they are not mutually exclusive and there are many papers that cover multiple keywords.
12. The clusters are derived from co-citation between papers and show only tendencies. Naturally, they are not mutually exclusive and there are citations between clusters.
13. It is important to stress that clusters are derived from keywords of papers. Because some papers may be interdisciplinary, include more than one topic, or combine different types of institutions, the resulting clusters are naturally interlinked and not necessarily mutually exclusive.
14. We have not included legal studies because it is more concerned with challenges of legal principles when national and supranational institutions collide, which is not our main focus.
15. As such, they can be conceived of as a hybrid form of supranational institution that combines formal and informal characteristics, depending on the national context. One example is the Sustainable Development Goals by the United Nations, which forms the basis of many national formal legislation and policies.
16. In political science, the idea of a transnational civil society interacting and influencing both national states and MNCs has a much longer tradition (Kaiser, 1971; Stopford, Strange, & Henley, 1991). Notable here are also the contributions by Donaldson & Dunfee (1994, 1999), which distinguish local norms of ethics from hypernorms which are akin to supranational institutions.
17. We distinguish the concept of institutional triality from a similarly named concept in Doh, Husted, & Marano (2019, p. 9), who refer to an institutional ‘triality’ that encompasses two co-existing (sub)nation-level institutional frames.
18. Sun et al. (2021) make a similar argument referring to this coexistence as institutional multiplicity.
19. A case documented in the literature is the case of the Rosia Montana gold mine in Romania in which European and US activists had a decisive role in the cancellation of the project after putting pressure on Romanian policy-makers (Henisz, 2011).
20. Scholarship in related fields has also referred to such institutional distances as schisms (Moore, Brandl, & Dau, 2021) or institutional abyss (Alcacer & Ingram, 2013).
21. Consequently, it may manifest even for organizations that are otherwise purely domestic.
22. Moore et al. (2021) provide a conceptual framework how compliance with supranational institutions may vary as a function of the national institutional context.
23. In addition to promoting opposing informal values and norms, national institutions may try to shield the country from supranational values through attempting to control the free flow of information (e.g., China).
24. From a bottom-up perspective, contextuality from opposing or inefficient national institutions is less salient because MNCs in a host country that do not support supranational institutions commonly can voluntarily adhere to higher-order norms.
25. In the context of international arbitration, for example, there is an ongoing and controversial discussion about the requirements for investments to enjoy protection under a specific national or international jurisdiction (Salini Criteria) (Sauvant, 2019).
26. Botzem & Dobusch (2012) provide case studies of the process of standard formation.
27. We recognize the importance of individuals as a grass-root tier but omit the individual level from the review and theory discussion in this paper.
28. Other blind spots within IB research identified in the bibliometric analysis, include, for example, the role of international sanctions (Meyer & Thein, 2014), the governing role of international financial markets (Dorobantu & Müllner, 2019), the role of MNCs in economic development, human rights, and democracy (Giuliani & Macchi, 2014).

## Supplementary Information

Below is the link to the electronic supplementary material.

Supplementary file1 (PDF 317 KB)
