# Supplementary material for: Beyond the nation-state: Anchoring supranational institutions in international business research
Source: J Int Bus Stud. 2022 Jun 27;53(6):1282–306. doi: 10.1057/s41267-022-00537-3 (PMC9244025; doi:10.1057/s41267-022-00537-3)
Supplement: Supplementary file 1 — Supplementary file1 (PDF 317 KB) [file 41267_2022_537_MOESM1_ESM.pdf]

BEYOND THE NATION-STATE: ANCHORING SUPRANATIONAL INSTITUTIONS IN INTERNATIONAL BUSINESS RESEARCH

ONLINE APPENDIX 1

We limit the analysis to keywords that are used at least 150 times in the body of 44,812 articles. This high threshold ensures that potential false positives (i.e., papers falsely identified as supranational and institutional by the search string) do not drive results, thereby ensuring an accurate representation of the "big topics" in the field.[1] Co-word analysis uses multiple correspondence analysis (MCA), an exploratory multivariate technique for the graphical and numerical analysis of multivariate categorical data (Abdi & Valentin, 2007). We conduct the analysis using the conceptualStructure() command in the Bibliometrix package (Aria & Cuccurullo, 2017). This command creates a matrix M, in which rows represent articles and columns represent all unique keywords. The procedure sets all elements of M (M_{ij}) to the value 1 when an article in row *j* uses a keyword listed in column *i.* All other entries are zero. Then, the package conducts an exploratory cluster analysis following the K means methodology to group keywords into clusters. The resulting output represents the clustering layer (i.e., the number of clusters) that maximizes the marginal variance explained per cluster. Figure 1 shows the output obtained from the co-word analysis.[2]

In terms of computational process, the co-word analysis proceeds as follows. First, we extract all keywords from the 44,812 articles obtained from the revised search string. We do so using the termExtraction() command in the Bibliometrix package (Aria & Cuccurullo, 2017) in R. We order them by frequency of mention. This function is supported by Porter's stemming algorithm to aggregate different forms of the same word.

Second, using the conceptualStructure() command, we use a Multiple Correspondence Analysis (MCA; Abdi & Valentin, 2007) algorithm to cluster the keywords:

a. First, the algorithm creates a matrix (M) with columns representing the 44,812 articles, and rows representing the unique (and reduced using the Porter (1980) algorithm) keywords.

b. Following the MCA logic, the package next normalizes M by dividing the values in M by the total count of occurrences of all keywords in all papers.
c. Then, MCA introduces two vectors that summarize the normalized M in terms of summing the normalized matrix M by row and by column.
d. Then, MCA computes a singular value decomposition of a derivative of the normalized matrix M.
e. This decomposition provides us factors for each keyword, representing the coordinates for each keyword in a two-dimensional space (which is what we plot in Figure 1 in the revised manuscript).

Building on this analysis, the conceptualStructure() command runs a k-means clustering algorithm, basing the distance metric on the coordinates the keywords have in Figure 1 in the main text. The clusters shown in Figure 1 are the highest layer of clustering, explaining the largest relative amount of variance with one clustering step.

APPENDIX 2

We use this information to build a raw co-citation matrix in which both columns and rows are represented by the 50 most cited works by the body of literature. In this square matrix **N**, the element $\mathbf{N}_{ij}$ shows the number of times work *i* and work *j* have been cited together. Diagonal elements represent the overall number of citations that a core article received in the body of literature. This raw co-citation matrix is then normalized and converted into a matrix of coefficients that represent the share of co-citations of documents divided by all citations of a document. Based on these coefficients, we perform a factor analysis to find main groups according to their similarity as expressed by (relative) co-citation frequencies. We conduct this analysis using the biblioNetwork() command in Bibliometrix (Aria & Cuccurullo, 2017) . Figure 2 shows the result of this co-citation analysis.

APPENDIX 3

First, we restrict the results to the three core disciplines of our review to management & business, economics and political science, which yields 22,287 papers (1,258 IB; 3,799 Econ; 17,230 PS) or roughly half the full body of literature. As these cover a broad range of topics and not all of them are relevant to IB, we add a second selection step, searching for research that includes IB relevant protagonists. For this purpose, we use the following search terms:[3] multinational company, multinational enterprise, transnational enterprise foreign direct investment, foreign investor, foreign firm, foreign business and international business. This second step leaves us with 632 papers (241 business & management, 163, economics, 228 political science) with MNC relevance. In the final step, we further reduce these 673 papers to 135 contributions, which we review in greater detail. The most central elements from these 135 contributions are included in the qualitative review.

Table A1: Literature selection

Discipline	Bibliometric Analysis	MNC-relevance	Considered for review
**Business & Management**	1,258	241	60
**Economics**	3,799	163	35
**Political Science**	17,230	228	36
**Other**	22,525	41	4
**Total**	44,812	673	135

Table A2: Top 5 Journals in the Qualitative Review by Discipline

Business & Management	Economics	Political Science
**Journal of International Business Studies (29)**	International Political Economy (12)	International Organization (10)
**Journal of Business Ethics (18)**	The Review of International Organization (6)	International Studies Quarterly (6)
**Journal of World Business (13)**	World Development (3)	The Journal of Conflict Resolution (5)
**International Business Review (10)**	Journal of International Economics (1)	The Annual Review of Political Science (3)
**Management International Review (9)**	Journal of Public Economics (1)	The Journal of Politics (4)

The results of this stepwise process reveal dominant subfields in the broader disciplines. Journal outlets in Table A2 show that the discipline of business & management articles is dominated by IB contributions whereas international economics and international political economy are

productive streams of research on supranational institutions in economics. Political science is dominated by international politics and international relations.

Finally and for quality assurance, we include an inclusion step. Specifically, we screen for highly important contributions among the 22,525 papers excluded in steps 1 and 2. This screening is based on citation counts and keywords and yields additional 41 papers with IB-related contributions, which we also read in detail to extract the central aspects for our qualitative review.[4] A table of the most recent related publications in JIBS is provided below in Table A3.

APPENDIX 4

Table A3: Relevant papers published recently in the Journal of International Business Studies

Albino-Pimentel, J., Oetzel, J., Oh, C. H., & Poggioli, N. A. (2021). Positive Institutional Changes Through Peace: The Relative Effects of Peace Agreements and Non-market Capabilities on FDI. *Journal of International Business Studies*, 52(7): 1256-78.
Buckley, P. J. (2021). The Theory and Empirics of the Structural Reshaping of Globalization. *Journal of International Business Studies*, 51: 1580–1592.
Doh, J. P., Budhwar, P., & Wood, G. (2021). Long-term Energy Transitions and International Business: Concepts, Theory, Methods, and a Research Agenda. *Journal of International Business Studies*, 52(5): 951-70.
Erin Bass, A., & Grøgaard, B. (2021) The Long-term Energy Transition: Drivers, Outcomes, and the Role of the Multinational Enterprise. *Journal of International Business Studies*, 52: 807–823.
Georgallis, P., Albino-Pimentel, J., & Kondratenko, N. (2021). Jurisdiction Shopping and Foreign Location Choice: The Role of Market and Nonmarket Experience in the European Solar Energy Industry. *Journal of International Business Studies*, 52(5): 853-77
Kano, L., Tsang, E. W., & Yeung, H. W. (2020). Global Value Chains: A Review of the Multi-Disciplinary Literature. *Journal of International Business Studies*, 51: 577–622.
Kostova, T., Beugelsdijk, S., Scott, W. R., Kunst, V. E., Chua, C. H., & van Essen, M. (2020). The Construct of Institutional Distance through the Lens of Different Institutional Perspectives: Review, Analysis, and Recommendations. *Journal of International Business Studies*, 51: 467-497.
Li, C., Arikan, I., Shenkar, O., & Arikan, A. (2020). The Impact of Country-dyadic Military Conflicts on Market Reaction to Cross-border Acquisitions, *Journal of International Business Studies*, 51(3): 299-325.
Minefee, I. & Bucheli, M. (2021). MNC Responses to International NGO Activist Campaigns: Evidence from Royal Dutch/Shell in Apartheid South *Africa. Journal of International Business Studies*, 52(5): 971-98.
Montiel, I., Cuervo-Cazurra, A., Park, J., Antolín-López, R., & Husted, B. W. (2021). Implementing the United Nations' Sustainable Development Goals in International Business. *Journal of International Business Studies*, 52(5): 999-1030.
Nippa, M., Patnaik, S., & Taussig, M. (2021). MNE Responses to Carbon Pricing Regulations: Theory and Evidence. *Journal of International Business Studies*, 52(5): 904-29.
Sun, P., Doh, J. P., Rajwani, T., & Siegel, D. (2021). Navigating Cross-border Institutional Complexity: A Review and Assessment of Multinational Nonmarket Strategy Research. *Journal of International Business Studies*, 52(1): 1818–1853
Tian, L., Tse, C. H., Xiang, X., Li, Y., & Pan, Y. (2021). Social Movements and International Business Activities of Firms. *Journal of International Business Studies*, 52(6): 1200-14

ENDNOTES

[1] Further manual quality checks highlighted the immense variety of papers, topics and disciplines but little reason to suspect bias from false positives.

[2] The clusters identified emerge from an exploratory analysis with very few adjustable parameters and remain robust under different specifications of the clustering algorithm. Details on the algorithm (and on MCA in general) are available in Abdi & Valentin (2007).

[3] Including singulars, plurals and their established abbreviations respectively.

[4] Specifically, (a) 20 citations or 5 citations per year, (b) published in a sub-discipline that includes either Business, Economics or Political Science, (c) explicit mention of an IB-related activity or actor (d) explicit reference to institutions in title or abstract.
